# Role of breastfeeding on maternal and childhood cancers: An umbrella review of meta-analyses

**DOI:** 10.7189/jogh.13.04067

**Published:** 2023-06-23

**Authors:** Dazhi Fan, Qing Xia, Dongxin Lin, Yubo Ma, Jiaming Rao, Li Liu, Hai Tang, Tingting Xu, Pengsheng Li, Gengdong Chen, Zixing Zhou, Xiaoling Guo, Zhifang Zhang, Zhengping Liu

**Affiliations:** 1 Foshan Fetal Medicine Research Institute, Affiliated Foshan Women and Children Hospital, Southern Medical University, Foshan, Guangdong, China; 2 Department of Obstetrics, Affiliated Foshan Women and Children Hospital, Southern Medical University, Foshan, Guangdong, China; 3 Australian Centre for Health Services Innovation and Centre for Healthcare Transformation, School of Public Health & Social Work, Faculty of Health, Queensland University of Technology, Australia; 4 Department of Epidemiology and Biostatistics, School of Public Health, Anhui Medical University, Hefei, Anhui, China; 5 Department of Library, the First Affiliated Hospital, College of Medicine, Zhejiang University, Hangzhou, Zhejiang, China; 6 Department of Health Management and Policy, School of Public Health, Capital Medical University, Beijing, China; 7 School of Medicine, Foshan University, Foshan, Guangdong, China

## Abstract

**Background:**

Multiple studies and meta-analyses have claimed that breastfeeding is inversely correlated with maternal and childhood cancers. These results could either be causal or confounded by shared risk factors. By conducting an umbrella review, we aimed to consolidate the relationship between breastfeeding and maternal and childhood cancers.

**Methods:**

We searched PubMed, Embase, Web of Science, Elsevier ScienceDirect, and Cochrane Library databases from inception to December 2022. Two reviewers independently extracted the data and assessed the quality of the studies using standardised forms. We considered two types of breastfeeding comparisons (“ever” vs “never” breastfeeding; and “longest” vs “shortest” duration). We estimated the pooled risk and 95% confidence interval (CI) for each meta-analysis.

**Results:**

We included seventeen meta-analyses with 55 comparisons. There was an inverse correlation between breastfeeding and childhood leukaemia (pooled risk = 0.90, 95% CI = 0.81-0.99), neuroblastoma (pooled risk = 0.81, 95% CI = 0.71-0.93), maternal ovarian cancer (pooled risk = 0.76, CI = 0.71-0.81), breast cancer (pooled risk = 0.85, 95% CI = 0.82-0.88), and oesophageal cancer (pooled risk = 0.67, 95% CI = 0.54-0.81) for “ever” vs “never” breastfeeding; and with childhood leukaemia (pooled risk = 0.94, 95% CI = 0.89-0.98), and maternal ovarian cancer (pooled risk = 0.84, 95% CI = 0.78-0.90) and breast cancer (pooled risk = 0.92, 95% CI = 0.89-0.96) for “longest” vs “shortest” breastfeeding duration.

**Conclusions:**

We found evidence that breastfeeding may reduce the risk of maternal breast cancer, ovarian cancers, and childhood leukaemia, suggesting positive implications for influencing women’s decision in breastfeeding.

**Registration:**

PROSPERO (CRD42021255608).

Cancer is a significant public health challenge, inflicting considerable health and economic strain on individuals, governments, and society [1]. It is the second leading cause of death worldwide, with an estimated 1.9 million new cancer cases and over 0.6 million cancer-related deaths in the USA in 2021 [2]. Nearly half of all new cancer cases and deaths occur among women and children [3,4]. Novel evidence highlighted the role of maternal reproductive health, apart from inherited genetic factors, in the risk of cancers prevalent in women and children [5,6]. Of these influences, breastfeeding, a modifiable reproductive factor, has emerged as a potentially consequential determinant in the development of certain cancer types. The absence or reduction of breastfeeding may disrupt the regulation of maternal endogenous oestrogens and DNA damage, as well as neonatal immune, anti-inflammatory, and antibacterial activity [7-9].

Universally recommended as the optimal nutritional source for newborn infants, breastfeeding is widely practised worldwide, and is especially relevant in low- and middle-income countries [7]. Accumulating evidence from multiple studies and meta-analyses highlighted the protective effect of breastfeeding against the risk of several maternal cancers, such as breast [10,11], ovarian [8,12], endometrial [13,14], and thyroid [9], and childhood cancers, including leukaemia [15,16], lymphoma [17,18], germ cell tumours [19], and neuroblastoma [17]. The relationship between breastfeeding and cancer risk is complex, potentially being either causal, confounded by common risk factors, or subject to research biases. While early observational studies suggested that breastfeeding could diminish the risk of endometrial cancer [20,21], more recent large-scale studies [22,23] and meta-analyses [24] have not supported these statements.

Given the substantial global burden of maternal and childhood cancers, it is necessary to understand the potential causal role of breastfeeding in cancer prevention. We conducted an umbrella review, examining the robustness of the evidence and the extent of potential bias in the relationship between breastfeeding and the risk of maternal and childhood cancers.

## METHODS

We prospectively registered the study on PROSPERO (CRD42021255608) and conducted it following the methodological guidance for conducting umbrella reviews in medicine [25-27]. Umbrella review is a next-generation evidence synthesis method, usually used to address a broader scope of research questions, providing a comprehensive and overarching summary of existing evidence [28-30].

### Literature search

We searched the PubMed, Embase, Web of Science, Elsevier ScienceDirect, and Cochrane Library databases from inception to December 2022 (without language restrictions) for meta-analyses of observational studies that investigated the correlations between breastfeeding and any maternal or childhood cancers. We develop the search strategy around key words: “breastfeeding”, “women”, “childhood”, “cancer”, “meta-analysis”, “systematic review” and their synonyms, limiting the results to systematic reviews and meta-analyses with a search filter (Text S1 in the **Online Supplementary Document**). We also manually searched the references of eligible systematic or narrative reviews. Two researchers (DF and LL) independently screened the titles and abstracts and selected the articles for full text review, resolving discrepancies by consensus with two other researchers (QX and DL).

### Study eligibility and selection

We managed the screening process in EndNote (version X7, Thomson ResearchSoft, Stanford, CA, USA). We included meta-analyses of individual observational studies (case-control (hospital-based or population-based), cohort, cross-sectional or ecological studies)) that examined the correlation between breastfeeding and maternal or childhood cancers, and studies that reported quantitative outcomes. We excluded studies that did not specifically include breastfeeding as an independent exposure.

If an article presented more than one eligible meta-analysis, we assessed them separately. Whenever more than one meta-analysis existed on the same research question, we applied the following criteria: if the primary studies were completely overlapping, we selected the one with higher GRADE quality [31]; if the primary studies did not overlap or partially overlapped, we selected the meta-analysis with the largest number of studies or the most recent one; if an article presented separate meta-analyses for more than one cancer type, we include each one separately.

### Data extraction

Two researchers (DF and YM) independently extracted the following data from eligible meta-analysis: first author, year of publication, number and type of studies included, comparison groups of breastfeeding, type of cancer, number of cancer cases/total number of participants, type of risk used for pooling (risk ratio (RR), odds ratio (OR), or hazard ratio (HR)), effect size and 95% confidence interval (CI), type of effect model used in the meta-analysis (fixed or random), and the largest effect size. We considered the two most performed comparison types in breastfeeding literature (i.e. “ever” vs “never” breastfeeding; the “longest” vs the “shortest” duration of breastfeeding). Per the included articles, we defined “ever” breastfeeding as any breastfeeding (regardless of duration), “never” breastfeeding as no breastfeeding history. We defined “longest duration of breastfeeding” as the total duration of breastfeeding lasting six months or longer and the “shortest” duration as having breastfed for less than six months overall. The detailed information on breastfeeding was self-reported. We also recorded when a meta-analysis considered a dose-response relation and published a *P*-value for nonlinearity (Table S1 in the **Online Supplementary Document**). Any difference in extracted data between the two researchers was resolved by consensus with two other researchers (QX and DL).

### Assessment of methodological quality of included studies

Two investigators (DF and YM) independently assessed the methodological quality of the included studies using the Assessment of multiple systematic reviews (AMSTAR) 2; validated through several studies, the tool categorises the quality of a meta-analysis on a scale from critically low to high, based on 16 predefined items [26,32,33] (Table S2 in the **Online Supplementary Document**).

### Data synthesis and analysis

We conducted all statistical analyses in STATA Software (version 12.0, StataCorp, College Station, Texas, USA). Units of analysis were the systematic reviews and meta-analyses meeting the inclusion criteria. For each meta-analysis, we re-calculated effect sizes, 95% CIs, and *P*-values to extract information on the original articles to evaluate the evidence level of meta-analyses through the inverse variance random-effects method. We simultaneously presented the results of the fixed-effects method. We recalculated the heterogeneity using the *I^2^* statistic and the *P*-value from the χ^2^-based Cochran Q test. We also estimated the 95% prediction interval (PI) for the summary random effects to further signify heterogeneity between studies and represent the accuracy of the summary effect size [34]. We assessed the evidence of small-study effects using the Egger regression test with a *P*-value <0.10. We constructed forest plots from the extracted and/or re-analysed data to display the two types of comparisons of breastfeeding for cancers (i.e. “ever” vs “never” breastfeeding, and the “longest” vs “shortest” breastfeeding duration), where available. We did not re-analyse the dose-response analysis due to the scarcity of corresponding data .

## RESULTS

### Study eligibility

**Figure 1:**
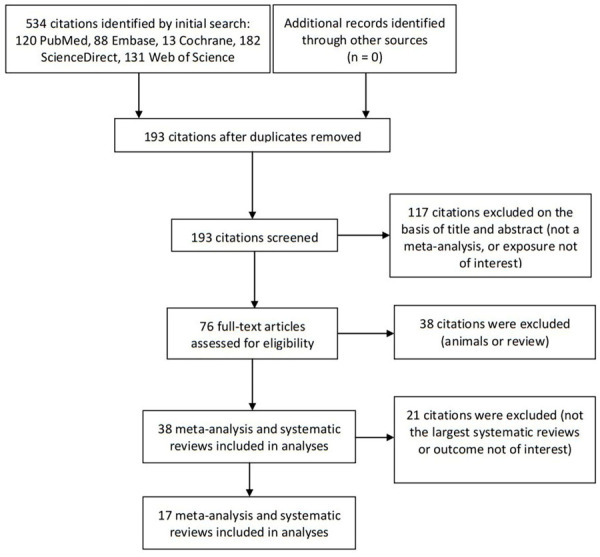
Flowchart of literature search.

We identified 534 articles identified from the databases, with 76 articles eligible for the full-text screening. After excluding ineligible studies, we included 17 meta-analyses with 55 comparisons [9,10,13,17-19,24,35-44] (**Figure 1**). The exclusion reasons for the 21 systematic reviews and meta-analyses are shown in Table S3 in the **Online Supplementary Document**.

### Description of meta-analyses

The 17 included meta-analyses covered associations of breastfeeding with risk of diagnosis from maternal breast, oesophageal, endometrial, thyroid, and ovarian, and childhood leukaemia, lymphoma, brains tumours, neuroblastoma, soft-tissue sarcoma, nephroblastoma, retinoblastoma, and germ cell tumours. These meta-analyses included 415 individual study estimates, with two to 93 study estimates combined per meta-analyses, with a median of nine studies. There was a median 3141 cases and a median total population of 50 555 in each meta-analysis, with the lowest number of cases in a meta-analysis being 126. Of the 415 individual studies included in this umbrella review, 375 (90.4%) were case-control design and 40 (9.6%) were cohort design. We found that nine types of cancer were reported in two or more meta-analysis: breast cancer (n = 15), ovarian cancer (n = 6), leukaemia (n = 5), endometrial cancer (n = 4), lymphoma (n = 3), oesophageal cancer (n = 2), brains tumours (n = 2), neuroblastoma (n = 2), soft-tissue sarcoma (n = 2), and germ cell tumours (n = 2).

### AMSTAR 2 classification of included studies

The detailed AMSTAR-2 assessment results for each meta-analysis are presented in Table S3 in the **Online Supplementary Document**. We rated only one meta-analysis as high and the others as low, primarily due to two factors: the inability to provide a protocol prior to the commencement of the review and the failure of review authors to employ a comprehensive literature search strategy.

### Summary effect size

**Figure 2:**
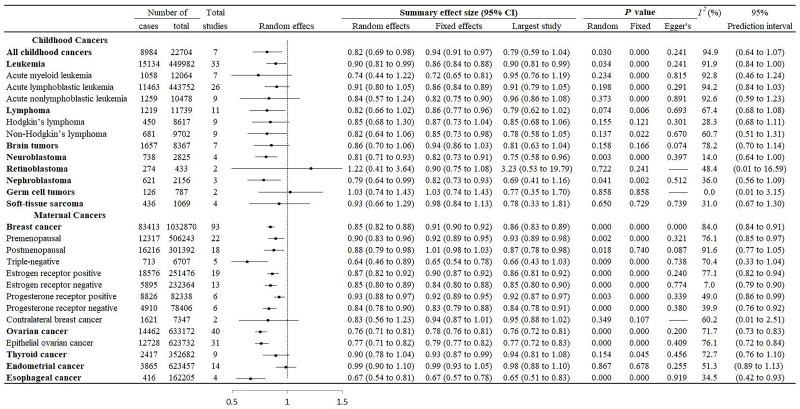
**“**Ever” vs “never” breastfeeding and associations with maternal and childhood cancers.

**Figure 2** shows the data for the meta-analysis of evidence for breastfeeding and women and childhood cancers for “ever” vs “never” comparison. We found a negative correlation between breastfeeding and childhood leukaemia and neuroblastoma, as well as maternal breast, ovarian, and oesophageal cancers. The pooled random effect size and PI were 0.90 (95% CI = 0.81-0.99, 95% PI = 0.84-1.00) for leukaemia, 0.81 (95% CI = 0.71-0.93, 95% PI = 0.64-1.00) for neuroblastoma, 0.85 (95% CI = 0.82-0.88, 95% PI = 0.84-0.91) for breast cancer, 0.76 (95% CI = 0.71-0.81, 95% PI = 0.73-0.83) for ovarian cancer, and 0.67 (95% CI = 0.54-0.81, 95% PI = 0.42-0.93) for oesophageal cancer,.

**Figure 3:**
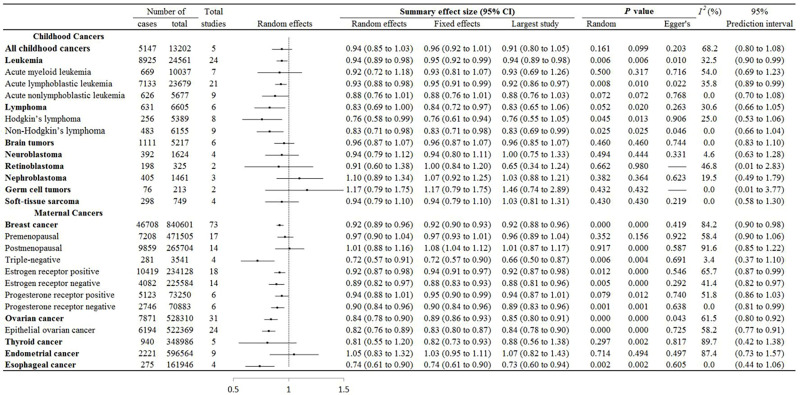
**“**Longest” vs “shortest” breastfeeding duration and associations with maternal and childhood cancers.

**Figure 3** shows summary data for the meta-analysis of evidence for breastfeeding and women and childhood cancers for the “longest” vs the “shortest” duration of breastfeeding. We found a negative correlation between breastfeeding and childhood leukaemia, and maternal breast and ovarian cancers. The pooled random effect size and prediction interval were 0.94 (95% CI = 0.89-0.98, 95% PI = 0.90-0.99) for leukaemia, 0.92 (95% CI = 0.89-0.96, 95% PI = 0.90-0.89) for breast cancer, and 0.84 (95% CI = 0.78-0.90, 95% PI = 0.80-0.92) for ovarian cancer.

We found the U-shaped curve for dose-response between breastfeeding and leukaemia using nonlinear dose-response analysis, and the risk of leukaemia was statistically significant at a duration of 4.4-15.0 months, with the most protective effect (0.66; 95% CI = 0.62-0.70) observed at a duration of 9.6 months [17]. There was also significant dose-response relation indicating benefit between breastfeeding and breast cancer [10], endometrial cancer [13,24], ovarian cancer [41,42], and thyroid cancer [9]. The dose-response analysis showed a decreasing nonlinear trend as the accumulated months of breastfeeding increased for breast cancer (*P* = 0.001) [10]. A linear dose response was apparent for endometrial cancer, with the risk decreased by 2% (0.98; CI = 0.97-0.99) [24] for every one-month and 7% (0.93; 95% CI = 0.88-0.97) [13] for every six-month increase in the duration of breastfeeding. There was some evidence for a linear dose-response, with the risk decreased by 2% (0.98; 95% CI = 0.97-0.99) [41] for every one-month for ovarian cancer and by 8% (0.92; 95% CI = 0.90-0.95) [42] for every five-month for epithelial ovarian cancer. Breastfeeding was a linear negative correlation with a risk of incident maternal thyroid cancer, decreasing by about 2% (0.98; 95% CI = 0.98-0.99) [9] for every increment of one month of breastfeeding. There was no significant estimate of risk of childhood lymphoma [17], childhood Hodgkin lymphoma [18], and childhood brain cancers [17] at any level of breastfeeding duration in nonlinear dose-response analysis. The results of the dose-response relationship were not reported for other cancers.

### Heterogeneity of included studies

We re-analysed the heterogeneity by random effects for comparing “ever” vs “never” and the “longest” vs “shortest” breastfeeding. About 52.7% (n/N = 29/55) of these had an *I*^2^>50%, with 64.3% (n = 18/28) for “ever” vs “never”, and 40.7% (n/N = 11/27) for the “longest” vs the “shortest” duration of breastfeeding (**Figure 2** and **Figure 3**).

### Publication bias of included studies

We performed Egger’s regression test for all cancer types in the two breastfeeding comparisons except for three cancers (i.e. contralateral breast cancer, and childhood retinoblastoma and germ cell tumours) due to the insufficient numbers (**Figure 2** and **Figure 3**). Of the re-analysed studies, five out of 50 had statistical evidence of publication bias. This included “ever” vs “never” comparisons for maternal breast cancer (*P* = 0.001), and the “longest” vs “shortest” comparisons for childhood leukaemia (*P*  = 0.010), acute lymphoblastic leukaemia (*P* = 0.022), non-Hodgkin’s lymphoma (*P* = 0.046), and maternal ovarian cancer (*P* = 0.043).

## DISCUSSION

Our findings suggest a potential inverse correlation between breastfeeding and the risk of certain maternal and childhood cancers, including, maternal, breast, and ovarian cancers, and childhood leukaemia. However, the results should be interpreted with caution due to various methodological challenges. While our dose-response results suggest a potential benefit from breastfeeding for leukaemia, the evidence is not entirely clear. The detected U-shaped curve may be an artefact of the modelling process or may reflect the impact of uncontrolled confounding factors, so further research is needed to confirm these results. The challenges involved in interpreting such complex data highlight the need for rigorous methodologies in future studies and careful consideration of potential confounding variables.

A possible relationship between breastfeeding and the risk of developing childhood cancers has long been speculated. Studies [17,19,45,46] have suggested that breastfeeding has preventive effects against several types of childhood cancers, including leukaemia, lymphoma, and germ cell tumours. Importantly, breast milk provides high levels of immunological, anti-inflammatory, and antimicrobial elements, all of which potentiate the anticancer activity of breastfed infant [7]. Furthermore, the infant gut microbiome can be directly modified through seeding from the maternal microbiome and the other effects of human milk [47]. Multiple studies have found that infant gut microbiome could potentially actively stimulate or modulate the immune system and promote its development early in life [48-50].

Breastfeeding was found to potentially improve mother-child bond and childhood cardiorespiratory fitness [51,52], which will consequently benefit both mother and child’s physical and mental health. Importantly, the aetiology and pathogenetic mechanisms are complex and largely unknown for most childhood cancers. For instance, as a malignant embryonal tumour of neural crest cells, it seems that early disruption of normal developmental processes, constitutional chromosomal rearrangements (16p12-13, 1p36, 11q14-23), abnormal expression of the neurotrophin receptors (NTRK1, NTRK2, and NTRK3 encoding TrkA, TrkB, and TrkC) and their ligands (NGF, BDNF, and neurotrophin-3), and some exposures in pregnancy are all strongly related to the pathogenetic mechanisms of neuroblastoma [53]. Therefore, additional mechanistic studies and more in-depth analyses focusing on molecular changes are needed.

The negative correlation between breastfeeding and the risk of specific maternal cancers can be explained by several biologically mechanisms, one being that breastfeeding can decrease the exposure the endogenous oestrogens, which may eventually decrease maternal cancers risk [9]. Another possible mechanism is that breastfeeding could help to eliminate cells with damaged DNA through the excretion of human milk, and further reduce susceptibility to mutations [8]. Additionally, breastfeeding can decrease serum concentrations of insulin in women, and further decrease serum concentrations of insulin-like growth factor IGF-1, which could affect proliferation and anti-apoptosis of malignant cells [10].

Findings from multiple meta-analysis studies indicate that longer periods of breastfeeding could lead to more reduction in the risk of cancers for women and child, such as breast cancer [10,35], ovarian cancer [8,38], thyroid cancer [9], endometrial cancer [13,24,36], leukaemia [17,39], and lymphoma [17,18], compared with occasional or shorter period of breastfeeding. Dose-response analyses of childhood leukaemia indicated that the largest risk reduction is related to breastfeeding duration of 9.6 months [17]; interestingly, an increase in duration beyond this time does not seem to be correlated with increased risk of harm. In maternal breast cancer, despite significant nonlinearity, relative risk reduced sequentially from breastfeeding duration from six to twelve months [38]. However, nonlinear link might simply be a modelling artefact, so it should be treated with caution. Results from this study suggested that long-term breastfeeding can reduce the risk of childhood leukaemia, and maternal ovarian and breast cancers, but not other maternal and childhood cancers, such as thyroid, endometrial, and lymphoma.

Many of the correlations between breastfeeding and cancers of women and children, largely found in cohort studies, could be affected by residual confounding. Empirical evidence suggests that confounding factors in observational relationships might be common in research on cancer epidemiology [54]. Age, body mass index, family history, menopausal status, number of births and abortions, contraceptive use, smoking, alcohol use for women, and gender, age, place of residence, ethnicity, maternal factors (age at birth, education, working status, smoking, alcohol use in pregnancy, and occupational exposure) are all related to breastfeeding and a considerable number of maternal and childhood cancers. These factors may indirectly or directly influence the relationship between breastfeeding and women and childhood cancers. Meanwhile, breastfeeding may be affected by factors such as higher education and income, which could improve the health of both mother and child.

Our umbrella review is affected by several confounding factors inherent in the included systematic reviews. Influences such as socioeconomic status, opportunities for cancer diagnosis, and disparities in the composition of covariables could potentially skew the observed correlations. Despite our thorough synthesis and analysis of the available evidence, the individual studies included in the meta-analyses might be subject to inherent biases and confounding elements. We have strived to account for these confounders through adjustments and sensitivity analyses where feasible, yet some residual confounding may persist. Moreover, our umbrella review is dependent on the methodology and data detailed in the included systematic reviews. In certain instances, the absence or insufficient reporting of specific details or data necessary for a thorough exploration and adjustment for confounding factors may restrict our ability to address potential biases. To counter these shortcomings, future primary research should present more detailed data on confounding factors and perform rigorous adjustments to minimise bias. Additionally, carrying out individual participant data meta-analyses or consolidating data across various studies could facilitate a more robust examination of confounding factors.

Another limitation is the lack of data to re-analyse the dose-response meta-analyses from the published articles. This dependence on published data presupposes the correctness of exposure and estimate data in the component studies. Differences in methods and modelling techniques used for dose-response analyses across the primary studies could introduce heterogeneity and affect the accuracy of the results. Furthermore, our review was exclusively centred on meta-analysis studies, possibly overlooking relevant systematic reviews without quantitative data. We also did not explore the relationship between breastfeeding and adult male cancers, which could be addressed by future studies.

This umbrella review has several strengths. We delivered a summary and evaluation of the evidence on the correlation between breastfeeding and the risk of cancers in women and children by incorporating 17 meta-analyses and accounting for 55 comparisons. Second, we thoroughly searched five large scientific literature databases. Two researchers independently selected and extracted the data from individual studies, which amplifies the dependability and authenticity of the results, as does the application of AMSTAR-2, a newly-revised and widely accepted instrument for gauging the quality of meta-analyses. We also utilised uniform methods like the inverse variance random-effects approach in the re-analysis of each meta-analysis, which improved the comparison and understanding of the results across diverse outcomes. We also computed indicators of publication bias and heterogeneity to further solidify our conclusions.

## CONCLUSIONS

Numerous meta-analyses have studied the effect of breastfeeding on several maternal or childhood cancers; most estimates yielded nominally significant results, with the risk of most cancers being lower in women and children with breastfeeding than those without. We carried out this umbrella review to re-analyse existing evidence and draw conclusions for the overall effects of breastfeeding on maternal and childhood cancers. We found that breastfeeding may have a protective effect regarding maternal breast and ovarian cancers, and childhood leukaemia. Our results may suggest a positive impact of women’s decision in breastfeeding, so we strongly suggest including the practice in public health.

## Additional material


Online Supplementary Document
