# Supplementary material for: Role of breastfeeding on maternal and childhood cancers: An umbrella review of meta-analyses
Source: J Glob Health. 2023 Jun 23;13:04067. doi: 10.7189/jogh.13.04067 (PMC10288171; doi:10.7189/jogh.13.04067)
Supplement: Online Supplementary Document [file jogh-13-04067-s001.pdf]

Supplementary files

Text S1. Search strategy used in the umbrella review. (P2)
Table S1. Dose-response relationship between breastfeeding and cancers. (P3)
Table S2. AMSTAR-2 results. (P4)
Table S3. Systematic reviews and meta-analyses that excluded from the umbrella review with reasons (n=21). (P6)

PRISMA Checklist. (P8)
MOOSE Checklist. (P11)

Text S1. Search strategy used in the umbrella review.

(breastfeeding OR “breast feeding” OR (breast AND feeding) OR lactation OR “infant feeding” OR “infant nutrition” OR “human milk” OR “breast milk” OR “exclusive breastfeeding” OR “exclusive breast feeding”) AND (child OR pediatric OR childhood OR children OR women OR woman OR “pregnancy woman” OR “pregnancy women” OR maternal) AND (cancer OR neoplasm OR neoplasia OR carcinoma OR tumor) AND (meta-analysis[Filter] OR systematic review[Filter])

Table S1. Dose-response relationship between breastfeeding and cancers.

Cancer type	Study	Results	Effect size (95%CI)	P value
Childhood leukemia	Su Q, 2021	U-shaped curve	0.66 (0.62-0.70) at 9.6 months	___
Maternal breast cancer	Unar-Munguia M, 2017	Non-linear decreasing	___	0.001
Maternal endometrial cancer	Wang L, 2015	Linear decreasing	0.98 (0.97-0.99) (2% decrease every one-month increase)	___
Maternal endometrial cancer	Ma X, 2018	Linear decreasing	0.93 (0.88-0.97) (7% decrease every six-month increase	___
Maternal ovarian cancer	Feng L, 2014	Linear decreasing	0.98 (0.97-0.99) (2% decrease every one-month increase)	0.001
Maternal epithelial ovarian cancer	Luan N, 2013	Linear decreasing	0.92 (0.90-0.95) (8% decrease every five-month increase)	___
Maternal thyroid cancer	Yi X, 2016	Linear decreasing	0.98 (0.98-0.99) (2% decrease every one-month increase)	___
Childhood lymphoma	Su Q, 2021	Non-dose-response relationship	___	0.05
Childhood Hodgkin lymphoma	Wang K, 2013	Non-dose-response relationship	___	0.44
Childhood brain cancers	Su Q, 2021	Non-dose-response relationship	___	0.77

Table S2. AMSTAR-2 results.

	AMSTAR-2*																
Study	Q1	Q2	Q3	Q4	Q5	Q6	Q7	Q8	Q9	Q10	Q11	Q12	Q13	Q14	Q15	Q16	Quality
Su Q, 2021	Y	Y	Y	Y	Y	Y	Y	Y	Y	Y	Y	Y	Y	Y	Y	Y	H
Akdeniz D, 2020	Y	N	Y	PY	Y	Y	Y	Y	Y	Y	Y	Y	Y	Y	Y	Y	L
Ma X, 2018	Y	N	Y	Y	Y	Y	Y	Y	Y	Y	Y	Y	Y	Y	Y	Y	L
Unar-Munguia M, 2017	Y	N	Y	Y	Y	Y	Y	Y	Y	Y	Y	Y	Y	Y	Y	Y	L
Zhu Y, 2017	Y	N	Y	Y	Y	Y	Y	Y	Y	Y	Y	Y	Y	Y	Y	Y	L
Yi X, 2016	Y	N	Y	PY	Y	Y	Y	Y	Y	Y	Y	Y	Y	Y	Y	Y	L
Zhou Y, 2015	Y	N	Y	PY	Y	Y	Y	Y	Y	Y	Y	Y	Y	Y	Y	Y	L
Islami F, 2015	Y	N	Y	Y	Y	Y	Y	Y	Y	Y	Y	Y	Y	Y	Y	Y	L
Chowdhury R, 2015	Y	N	Y	Y	Y	Y	Y	Y	Y	Y	Y	Y	Y	Y	Y	Y	L
Zhan B, 2015	Y	N	Y	Y	Y	Y	Y	Y	Y	Y	Y	Y	Y	Y	Y	Y	L
Wang L, 2015	Y	N	Y	Y	Y	Y	Y	Y	Y	Y	Y	Y	Y	Y	Y	Y	L
Amitay EL, 2015	Y	N	Y	Y	Y	Y	Y	Y	Y	Y	Y	Y	Y	Y	Y	Y	L
Li DP, 2014	Y	N	Y	Y	Y	Y	Y	Y	Y	Y	Y	Y	Y	Y	Y	Y	L
Feng LP, 2014	Y	N	Y	Y	Y	Y	Y	Y	Y	Y	Y	Y	Y	Y	Y	Y	L
Luan NN, 2013	Y	N	Y	PY	Y	Y	Y	Y	Y	Y	Y	Y	Y	Y	Y	Y	L
Wang KL, 2013	Y	N	Y	Y	Y	Y	Y	Y	Y	Y	Y	Y	Y	Y	Y	Y	L
Martin RM, 2005	Y	N	Y	PY	Y	Y	Y	Y	Y	Y	Y	Y	Y	Y	Y	Y	L

Footnote:

Q1: Did the research questions and inclusion criteria for the review include the components of PICO?

Q2: Did the report of the review contain an explicit statement that the review methods were established prior to the conduct of the review and did the report justify any significant deviations from the protocol?

Q3: Did the review authors explain their selection of the study designs for inclusion in the review?

Q4: Did the review authors use a comprehensive literature search strategy?

Q5: Did the review authors perform study selection in duplicate?

Q6: Did the review authors perform data extraction in duplicate?

Q7: Did the review authors provide a list of excluded studies and justify the exclusions?

Q8: Did the review authors describe the included studies in adequate detail?

Q9: Did the review authors use a satisfactory technique for assessing the risk of bias (RoB) in individual studies that were included in the review?

Q10: Did the review authors report on the sources of funding for the studies included in the review?

Q11: If meta-analysis was performed did the review authors use appropriate methods for statistical combination of results?

Q12: If meta-analysis was performed, did the review authors assess the potential impact of RoB in individual studies on the results of the meta-analysis or other evidence synthesis?

Q13: Did the review authors account for RoB in individual studies when interpreting/discussing the results of the review?

Q14: Did the review authors provide a satisfactory explanation for, and discussion of, any heterogeneity observed in the results of the review?

Q15: If they performed quantitative synthesis did the review authors carry out an adequate investigation of publication bias (small study bias) and discuss its likely impact on the results of the review?

Q16: Did the review authors report any potential sources of conflict of interest, including any funding they received for conducting the review?

* AMSTAR-2 items do not only give an overall score, but also an overall rating based on weaknesses in the critical domains. According to the degree of conformity of the evaluation standard, it is evaluated as “Yes”, “Partial Yes” and “No”. When the evaluation is yes “1 point for "yes", 0 points for "no" and 0 points for "partial yes". Of the16 items of AMSTAR 2, seven items (**Q2, Q4, Q7, Q9, Q11, Q12, and Q15**) play important roles in the production of systematic reviews and the validity of results. AM-STAR-2 classifies the overall confidence on the results of the review into four levels: 1) **High:** No or one non-critical weakness – the systematic review provides an accurate and comprehensive summary of the results of the available studies that address the question of interest; 2) **Moderate:** More than one non-critical weakness – the systematic review has more than one weakness but no critical flaws. It may provide an accurate summary of the results of the available studies that were included in the review; 3) **Low:** One critical flaw with or without non-critical weaknesses – the review has a critical flaw and may not provide an accurate and comprehensive summary of the available studies that address the question of interest; 4) **Critically low:** More than one critical flaw with or without non-critical weaknesses – the review has more than one critical flaw and should not be relied on to provide an accurate and comprehensive summary of the available studies.

Table S3. Systematic reviews and meta-analyses that excluded from the umbrella review with reasons (n=21)

**References**	**Reasons for exclusion**
Han MA[1]	Breastfeeding is not involved.
Alipour S[2]	Outcome is not interest.
Gungor D[3]	It is not the largest studies.
Wang BJ[4]	It is not the latest research.
Sung HK[5]	It is not the largest studies.
Anothaisintawee T[6]	It is not the largest studies.
Nagata C[7]	It is not the largest studies.
do Carmo Franca-Botelho A[8]	It is a review, and the data is not provided.
Cohen JM[9]	It is not the largest studies.
Yang L[10]	It is not the largest studies.
Martin RM[11]	It is not the largest studies.
Kwan ML[12]	It is not the largest studies.
Cancer CGoHFiB[13]	It is not the largest studies.
Investigators UKCCS[14]	It is not the largest studies.
Bernier MO[15]	It is not the largest studies.
Shamshirian A[16]	It is not the largest studies.
Ma H[17]	It is not the latest studies.
Lambertini M[18]	It is not the latest studies.
Jordan SJ[19]	It is not the latest studies.
Babic A[20]	It is not the largest studies.
Nichols HB[21]	It is not the largest studies.

References

1. Han MA, Storman D, Al-Rammahy H, et al. Impact of maternal reproductive factors on cancer risks of offspring: A systematic review and meta-analysis of cohort studies. *PloS one.* 2020;15(3):e0230721.
2. Alipour S, Eskandari A, Johar FM, Furuya S. Phyllodes Tumor of the Breast during Pregnancy and Lactation; A Systematic Review. *Archives of Iranian medicine.* 2020;23(7):488-497.
3. Gungor D, Nadaud P, Dreibelbis C, et al. Infant milk-feeding practices and childhood leukemia: a systematic review. *The American journal of clinical nutrition.* 2019;109(Suppl_7):757S-771S.
4. Wang BJ, Zhang B, Yan SS, et al. Hormonal and reproductive factors and risk of esophageal cancer in women: a meta-analysis. *Diseases of the esophagus : official journal of the International Society for Diseases of the Esophagus.* 2016;29(5):448-454.
5. Sung HK, Ma SH, Choi JY, et al. The Effect of Breastfeeding Duration and Parity on the Risk of Epithelial Ovarian Cancer: A Systematic Review and Meta-analysis. *Journal of preventive medicine and public health = Yebang Uihakhoe chi.* 2016;49(6):349-366.
6. Anothaisintawee T, Wiratkapun C, Lerdsitthichai P, et al. Risk Factors of Breast Cancer: A Systematic Review and Meta-Analysis. *Asia Pacific Journal of Public Health.*

2013;25(5):368-387.

7. Nagata C, Mizoue T, Tanaka K, et al. Breastfeeding and breast cancer risk: an evaluation based on a systematic review of epidemiologic evidence among the Japanese population. *Japanese journal of clinical oncology.* 2012;42(2):124-130.
8. do Carmo Franca-Botelho A, Ferreira MC, Franca JL, Franca EL, Honorio-Franca AC. Breastfeeding and its relationship with reduction of breast cancer: a review. *Asian Pacific journal of cancer prevention : APJCP.* 2012;13(11):5327-5332.
9. Cohen JM, Hutcheon JA, Julien SG, Tremblay ML, Fuhrer R. Insufficient milk supply and breast cancer risk: a systematic review. *PloS one.* 2009;4(12):e8237.
10. Yang L, Jacobsen KH. A Systematic Review of the Association between Breastfeeding and Breast Cancer. *Journal of Women's Health.* 2008;17(10):1635-1645.
11. Martin RM, Middleton N, Gunnell D, Owen CG, Smith GD. Breast-feeding and cancer: the Boyd Orr cohort and a systematic review with meta-analysis. *Journal of the National Cancer Institute.* 2005;97(19):1446-1457.
12. Kwan ML, Buffler PA, Abrams B, Kiley VA. Breastfeeding and the risk of childhood leukemia: a meta-analysis. *Public health reports.* 2004;119(6):521-535.
13. Cancer CGoHFiB. Breast cancer and breastfeeding: collaborative reanalysis of individual data from 47 epidemiological studies in 30 countries, including 50 302 women with breast cancer and 96 973 women without the disease. *The Lancet.* 2002;360(9328):187-195.
14. Investigators UKCCS. Breastfeeding and childhood cancer. *British journal of cancer.* 2001;85(11):1685-1694.
15. Bernier MO, Plu-Bureau G, Bossard N, Ayzac L, Thalabard JC. Breastfeeding and risk of breast cancer: a metaanalysis of published studies. *Human reproduction update.* 2000;6(4):374-386.
16. Shamshirian A, Heydari K, Shams Z, et al. Breast cancer risk factors in Iran: a systematic review & meta-analysis. *Hormone molecular biology and clinical investigation.* 2020;41(4).
17. Ma H, Bernstein L, Pike MC, Ursin G. Reproductive factors and breast cancer risk according to joint estrogen and progesterone receptor status: a meta-analysis of epidemiological studies. *Breast cancer research : BCR.* 2006;8(4):R43.
18. Lambertini M, Santoro L, Del Mastro L, et al. Reproductive behaviors and risk of developing breast cancer according to tumor subtype: A systematic review and meta-analysis of epidemiological studies. *Cancer treatment reviews.* 2016;49:65-76.
19. Jordan SJ, Na R, Johnatty SE, et al. Breastfeeding and Endometrial Cancer Risk: An Analysis From the Epidemiology of Endometrial Cancer Consortium. *Obstetrics and gynecology.* 2017;129(6):1059-1067.
20. Babic A, Sasamoto N, Rosner BA, et al. Association Between Breastfeeding and Ovarian Cancer Risk. *JAMA oncology.* 2020;6(6):e200421.
21. Nichols HB, Schoemaker MJ, Cai J, et al. Breast Cancer Risk After Recent Childbirth: A Pooled Analysis of 15 Prospective Studies. *Annals of internal medicine.* 2019;170(1):22-30.

Section/Topic	#	Checklist Item	Reported on Page #
**TITLE**			
Title	1	Identify the report as a systematic review, meta-analysis, or both.	1
**ABSTRACT**			
Structured summary	2	Provide a structured summary including, as applicable: background; objectives; data sources; study eligibility criteria, participants, and interventions; study appraisal and synthesis methods; results; limitations; conclusions and implications of key findings; systematic review registration number.	2
**INTRODUCTION**			
Rationale	3	Describe the rationale for the review in the context of what is already known.	3
Objectives	4	Provide an explicit statement of questions being addressed with reference to participants, interventions, comparisons, outcomes, and study design (PICOS).	3
**METHODS**			
Protocol and registration	5	Indicate if a review protocol exists, if and where it can be accessed (e.g., Web address), and, if available, provide registration information including registration number.	4
Eligibility criteria	6	Specify study characteristics (e.g., PICOS, length of follow-up) and report characteristics (e.g., years considered, language, publication status) used as criteria for eligibility, giving rationale.	4
Information sources	7	Describe all information sources (e.g., databases with dates of coverage, contact with study authors to identify additional studies) in the search and date last searched.	4
Search	8	Present full electronic search strategy for at least one database, including any limits used, such that it could be repeated.	4
Study selection	9	State the process for selecting studies (i.e., screening, eligibility, included in systematic review, and, if applicable, included in the meta-analysis).	4

Data collection process	10	Describe method of data extraction from reports (e.g., piloted forms, independently, in duplicate) and any processes for obtaining and confirming data from investigators.	5
Data items	11	List and define all variables for which data were sought (e.g., PICOS, funding sources) and any assumptions and simplifications made.	5
Risk of bias in individual studies	12	Describe methods used for assessing risk of bias of individual studies (including specification of whether this was done at the study or outcome level), and how this information is to be used in any data synthesis.	5
Summary measures	13	State the principal summary measures (e.g., risk ratio, difference in means).	5
Synthesis of results	14	Describe the methods of handling data and combining results of studies, if done, including measures of consistency (e.g., I^2) for each meta-analysis.	5

Section/Topic	#	Checklist Item	Reported on Page #
Risk of bias across studies	15	Specify any assessment of risk of bias that may affect the cumulative evidence (e.g., publication bias, selective reporting within studies).	5
Additional analyses	16	Describe methods of additional analyses (e.g., sensitivity or subgroup analyses, meta-regression), if done, indicating which were pre-specified.	5
**RESULTS**			
Study selection	17	Give numbers of studies screened, assessed for eligibility, and included in the review, with reasons for exclusions at each stage, ideally with a flow diagram.	6
Study characteristics	18	For each study, present characteristics for which data were extracted (e.g., study size, PICOS, follow-up period) and provide the citations.	6
Risk of bias within studies	19	Present data on risk of bias of each study and, if available, any outcome level assessment (see item 12).	6

Results of individual studies	20	For all outcomes considered (benefits or harms), present, for each study: (a) simple summary data for each intervention group (b) effect estimates and confidence intervals, ideally with a forest plot.	7
Synthesis of results	21	Present the main results of the review. If meta-analyses done, include for each, confidence intervals and measures of consistency.	7
Risk of bias across studies	22	Present results of any assessment of risk of bias across studies (see Item 15).	8
Additional analysis	23	Give results of additional analyses, if done (e.g., sensitivity or subgroup analyses, meta-regression [see Item 16]).	8
**DISCUSSION**			
Summary of evidence	24	Summarize the main findings including the strength of evidence for each main outcome; consider their relevance to key groups (e.g., healthcare providers, users, and policy makers).	8
Limitations	25	Discuss limitations at study and outcome level (e.g., risk of bias), and at review-level (e.g., incomplete retrieval of identified research, reporting bias).	10
Conclusions	26	Provide a general interpretation of the results in the context of other evidence, and implications for future research.	10
**FUNDING**			
Funding	27	Describe sources of funding for the systematic review and other support (e.g., supply of data); role of funders for the systematic review.	11

From: Moher D, Liberati A, Tetzlaff J, Altman DG, The PRISMA Group (2009). Preferred Reporting Items for Systematic Reviews and Meta-Analyses: The PRISMA Statement. PLoS Med 6(6): e1000097. doi:10.1371/journal.pmed1000097

For more information, visit: **www.prisma-statement.org**.

MOOSE Checklist for Meta-analyses of Observational Studies

Item No	Recommendation	Reported on Page No
Reporting of background should include		
1	Problem definition	3
2	Hypothesis statement	3
3	Description of study outcome(s)	3
4	Type of exposure or intervention used	3
5	Type of study designs used	3
6	Study population	3
Reporting of search strategy should include		
7	Qualifications of searchers (eg, librarians and investigators)	4
8	Search strategy, including time period included in the synthesis and key words	4
9	Effort to include all available studies, including contact with authors	4
10	Databases and registries searched	4
11	Search software used, name and version, including special features used (eg, explosion)	4
12	Use of hand searching (eg, reference lists of obtained articles)	4
13	List of citations located and those excluded, including justification	4
14	Method of addressing articles published in languages other than English	4
15	Method of handling abstracts and unpublished studies	4
16	Description of any contact with authors	4
Reporting of methods should include		
17	Description of relevance or appropriateness of studies assembled for assessing the hypothesis to be tested	4
18	Rationale for the selection and coding of data (eg, sound clinical principles or convenience)	4
19	Documentation of how data were classified and coded (eg, multiple raters, blinding and interrater reliability)	5
20	Assessment of confounding (eg, comparability of cases and controls in studies where appropriate)	5
21	Assessment of study quality, including blinding of quality assessors, stratification or regression on possible predictors of study results	5
22	Assessment of heterogeneity	5
23	Description of statistical methods (eg, complete description of fixed or random effects models, justification of whether the chosen models account for predictors of study results, dose-response models, or cumulative meta-analysis) in sufficient detail to be replicated	5
24	Provision of appropriate tables and graphics	6
Reporting of results should include		
25	Graphic summarizing individual study estimates and overall estimate	6
26	Table giving descriptive information for each study included	6
27	Results of sensitivity testing (eg, subgroup analysis)	7
28	Indication of statistical uncertainty of findings	7

Item No	Recommendation	Reported on Page No
Reporting of discussion should include		
29	Quantitative assessment of bias (eg, publication bias)	8
30	Justification for exclusion (eg, exclusion of non-English language citations)	8
31	Assessment of quality of included studies	8
Reporting of conclusions should include		
32	Consideration of alternative explanations for observed results	10
33	Generalization of the conclusions (ie, appropriate for the data presented and within the domain of the literature review)	10
34	Guidelines for future research	10
35	Disclosure of funding source	11

From: Stroup DF, Berlin JA, Morton SC, et al, for the Meta-analysis Of Observational Studies in Epidemiology (MOOSE) Group. Meta-analysis of Observational Studies in Epidemiology. A Proposal for Reporting. *JAMA*. 2000;283(15):2008-2012. doi: 10.1001/jama.283.15.2008.

Transcribed from the original paper within the NEUROSURGERY® Editorial Office, Atlanta, GA, United Sates. August 2012.
